# Supplementary material for: Intrinsically flexible multimode reconfigurable transistors for polymorphic circuits and neuromorphic devices
Source: Nat Commun. 2025 Dec 9;17:333. doi: 10.1038/s41467-025-67041-8 (PMC12789138; doi:10.1038/s41467-025-67041-8)
Supplement: Supplementary file 1 — Supplementary Information [file 41467_2025_67041_MOESM1_ESM.pdf]

Supplementary Information for

Intrinsically Flexible Multimode Reconfigurable Transistors for Polymorphic Circuits and Neuromorphic Devices

Wanting Wang[1,2], Rui Qiu[2], Jiahao Zhu[2], Tianyu Zhu[2], Jialiang Wang[3], Dexing Liu[1], Jiaqiao Liang[1], Chunxiu Wang[1,2], Sixin Zhang[2], Zifan Wang[2], Qiuyue Huang[2], Xinwei Wang[3], Min Zhang[1,2,*]
[1] School of Science and Engineering, The Chinese University of Hong Kong, Shenzhen, 518172, China.
[2] School of Electronic and Computer Engineering, Peking University, Shenzhen, 518055, China.
[3]School of Advanced Materials, Peking University, Shenzhen 518055, China.
[*]Email: mzhang@cuhk.edu.cn

Supplementary Table 1. Summary of state-of-the-art reconfigurable transistors featuring both n-type and p-type operation modes.

Ref.	Substrate	Demostration
S1	Si	polarity-switchable diodes, memory, heterosynaptic plasticity and in-memory logic
S2	Si	reversible schottky rectifier, reconfigurable photovoltaic performance
S3	Si	artificial synapse, circuits: AND/OR, NAND/NOR and XOR/XNOR gate
S4	Si	circuits: 2:1 multiplexer, D-latch, 1-bit full adder and subtractor, synaptic circuits based on three devices
S5	Si	memory, circuits: linear and nonlinear logic gates with in situ storage, half-adder
S6	Si	circuits: NAND, NOR, and XOR
S7	Si	circuits: XOR, NAND, NOR, and buffers
S8	Si	circuits: a security primitive circuit with polymorphic NAND/NOR obfuscation functionality
S9	Si	circuits: transformable logic gates, reconfigurable half adder/subtractor
S10	Si	circuits: half-wave rectification, multivalued logic circuits,
S11	PDMS (flexible)	stretchable reconfigurable artificial synapses
S12	Si	reconfigurable artificial synapses
S13	Si	reconfigurable artificial synapses
S14	Glass	reconfigurable artificial synapses
S15	Si	reconfigurable artificial synapses
**This work**	PEN (flexible)	reconfigurable artificial synapses and dendrite integration, circuits: polymorphic circuits (inverters and NAND/NOR)

Supplementary Note 1. The state-of-the-art related to "reconfigurable transistors" and "flexible reconfigurable transistors".

Supplementary Table 1 summarizes state-of-the-art reconfigurable transistors featuring both n-type and p-type operation modes. The comparative analysis primarily focuses on their flexibility and demonstration. To the best of our knowledge, flexible reconfigurable transistors have only been reported in Ref. S11 thus far. Among these studies, most concentrate solely on either circuit implementations or artificial synapse applications, with very few demonstrating capabilities across both representative domains. Compared with these previous studies, our work has realized flexible reconfigurable transistors with demonstrations in circuits, artificial synapses and dendrite integration, which significantly enhances its appeal to a broader research community.

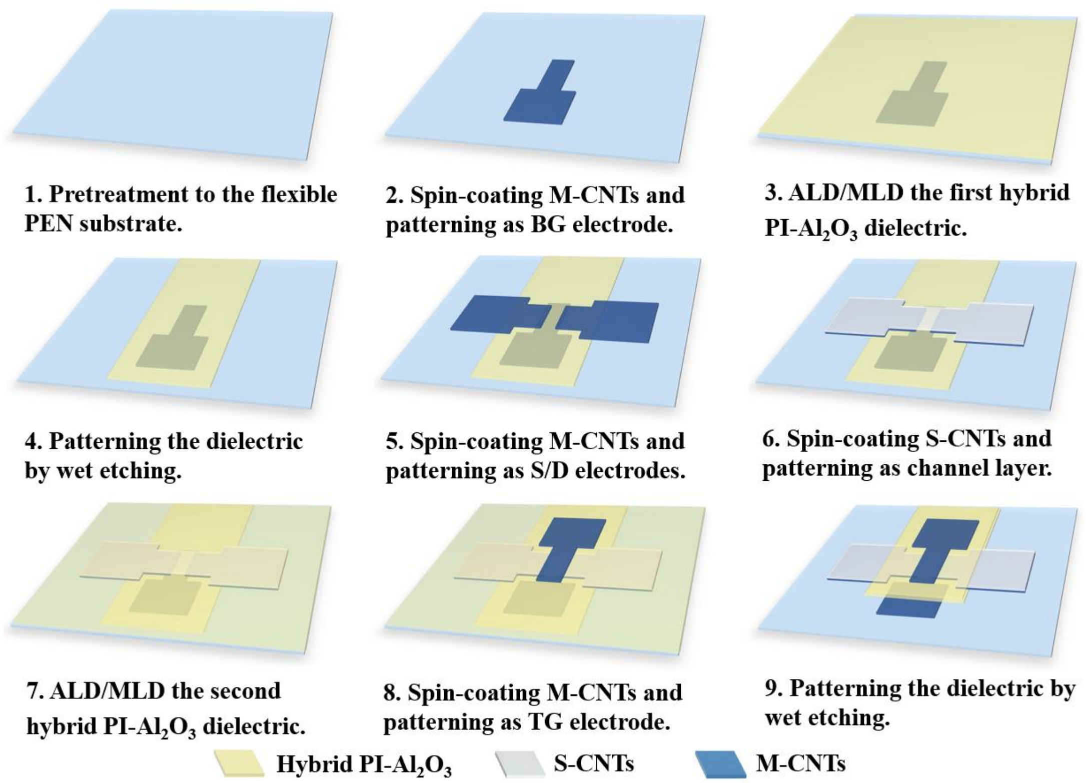

Supplementary Figure 1. Fabrication process flow of a typical IFMRTs.

Supplementary Table 2. Precise optical transmittance values for each individual layer and the complete IFMRT array at 500 nm, 700 nm and 900 nm under both "with substrate" and "without substrate" conditions.

		500nm(%)	700nm(%)	800nm(%)
**M-CNT Gate layer**	w/o substrate	95.2	97.1	98.9
	w/i substrate	91.7	97.0	97.6
**S-CNT Channel layer**	w/o substrate	97.0	96.9	97.9
	w/i substrate	92.2	96.8	97.7
**M-CNT S/D layer**	w/o substrate	94.4	97.1	98.7
	w/i substrate	91.8	95.8	97.3
**Hybrid PI-Al_2O_3 Dielectric layer**	w/o substrate	97.2	97.7	99.0
	w/i substrate	96.2	96.4	97.5
**IFMRT array**	w/o substrate	77.4	80.6	81.2
	w/i substrate	63.2	69.6	71.6

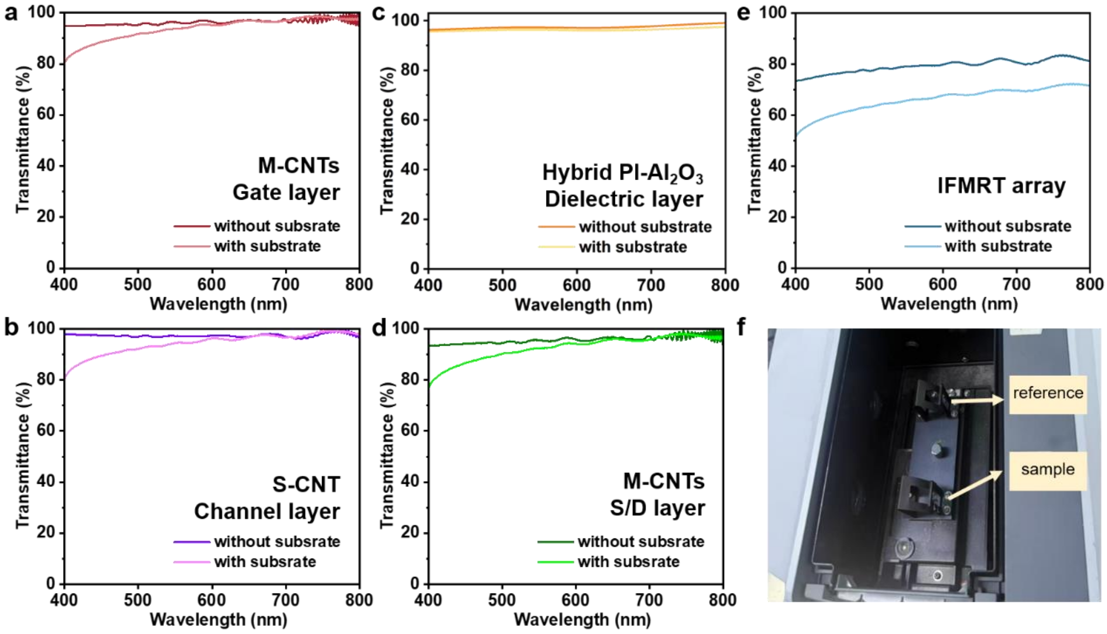

Supplementary Figure 2. Optical transmittance of IFMRTs and each individual layer in the visible light range. (a-e) The optical transmittance of each layer (a-d) and the IFMRT array (e) across the visible light spectrum. **(f)** UV-2600 spectrophotometer from SHIMADZU CORPORATION used to measure optical transmittance.

Supplementary Note 2. The clarification of the optical transmittance test.

As illustrated in Supplementary Fig. 2f, the spectrophotometer employs a dual-fixture configuration: one for the reference and the other for the test sample. The “with substrate” means that the transmittance was measured without any reference. The “without substrate” means that we used a blank substrate as reference and measured the substrate-normalized transmittance.

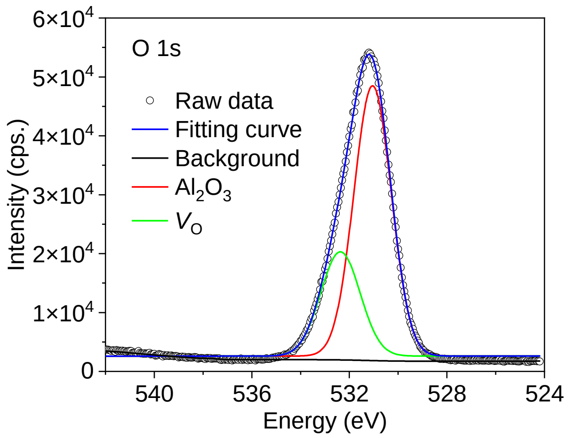

Supplementary Figure 3. The spectra by X-ray Photoelectron Spectroscopy (XPS) of Al_2O_3 film deposited by atomic layer deposition (ALD).

Supplementary Note 3. Scientific explanation of n-type doping.

CNT transistors typically exhibit p-type conducting properties mainly due to hole doping induced by water-oxygen reduction pairs in the environment [S16-17]. In this work, the high-temperature and high-vacuum environment during ALD deposition of Al_2O_3 isolates water and oxygen in the environment thus weakening the p-type doping effect. Besides, positively charged oxygen vacancies in ALD deposited Al_2O_3 can result in the n-type electrostatic doping effect on the CNT channel [S18]. We characterized the X-ray Photoelectron Spectroscopy (XPS) of the ALD deposited Al_2O_3 film in this work, as shown in Supplementary Fig. 3, indicating the presence of oxygen vacancies in IFMRTs and potential n-type electrostatic doping effect.

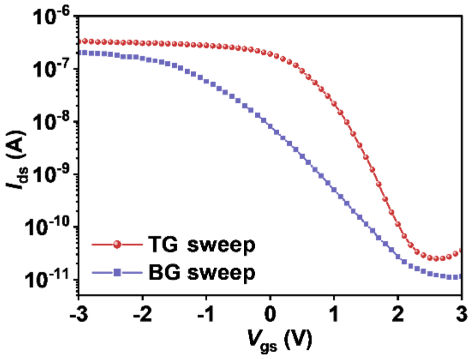

Supplementary Figure 4. Transfer characteristics of the IFMRT when the channel switching is respectively swept by TG (red) or BG (blue). (V_{ds}=−0.1 V, W=50 μm, L=50 μm)

Supplementary Note 4. The detailed scientific explanation of coupling strength, *SS* and interface states.

BG is set to 0 V under TG sweep, and TG is set to 0 V under BG sweep. During the fabrication of IFMRTs, the S/D electrodes are processed after the deposition of the bottom dielectric layer. When etching the S/D electrodes using oxygen plasma, the surface of the bottom dielectric layer in the channel region between the S/D electrodes is exposed to the plasma. This introduces interface states between the channel and the bottom dielectric, affecting transistor performance, particularly the subthreshold swing (*SS*). The relationship between *SS* and the interface state density (N_{it}) can be expressed by the following equation:

$$SS = \ln 10 \cdot \frac{dV_{GS}}{d(\ln I_{DS})} = \ln 10 \frac{kT}{q}\left(1 + \frac{C_D + qN_{it}}{C_{ox}}\right), \quad (1)$$

where k is the Boltzmann constant, T is the absolute temperature, q is the unit electron charge, C_D is the semiconductor depletion capacitance per unit area, and C_{OX} is the gate dielectric capacitance per unit area. Both the top and bottom dielectric layers have identical thicknesses of 38 nm, yielding similar C_{OX} values, about 125 nF·cm^{-2} at 335 Hz. The oxygen plasma treatment introduces additional interface states between the bottom dielectric layer and the channel, increasing N_{it}, thereby increasing *SS* and weakening the coupling capability of BG. In contrast, the top dielectric layer is fabricated by direct atomic layer deposition (ALD) and molecular layer deposition (MLD) on the channel without the effect of oxygen plasma, resulting in superior interface with channel. Therefore, *SS* during TG sweeping is smaller than that during BG sweeping, as shown in the transfer characteristics in Supplementary Fig. 4.

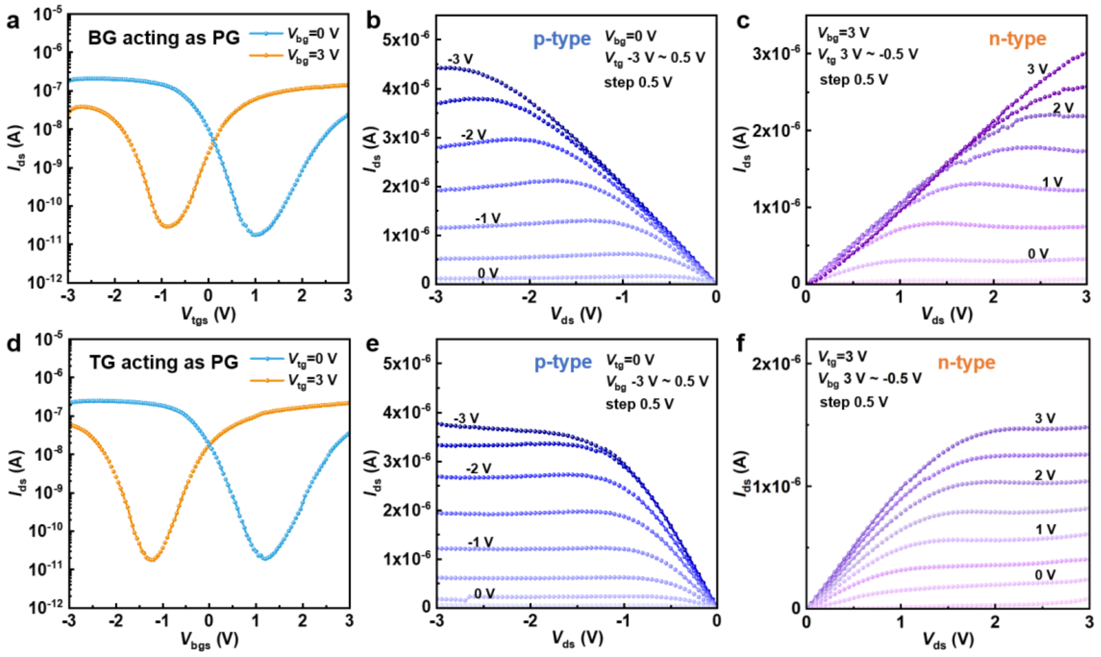

Supplementary Figure 5. Transfer characteristics and output characteristics of p-type and n-type IFMRTs. (a-c) Transfer characteristics (V_{ds}=−0.1 V, W=50 μm, L=50 μm) (a) and output characteristics of p-type (b) and n-type (c) IFMRTs when BG acting as PG and set to 0 V and 3 V. **(d-f)** Transfer characteristics (V_{ds}=−0.1 V, W=50 μm, L=50 μm) (d) and output characteristics of p-type (e) and n-type (f) IFMRTs when TG acting as PG and set to 0 V and 3 V.

Supplementary Note 5. Saturation current and the calculations of output resistance and the intrinsic gain.

When BG acting as PG, the saturation current is 4.42 μA in p-type mode under the condition of "V_{ds}=-3 V" and "V_{gs}=-3 V" (Supplementary Fig. 5b). The saturation current is 3.01 μA in n-type mode under the condition of "V_{ds}=3 V" and "V_{gs}=3 V" (Supplementary Fig. 5c). When TG acting as PG, the saturation current values are 3.77 μA and 1.48 μA in p-type (Supplementary Fig. 5e) and n-type mode (Supplementary Fig. 5f), respectively.

We also calculated the output resistance r_o. When BG acting as PG, in p-type mode (Supplementary Fig. 5b), under the condition of "V_{gs}=-3 V" and "V_{ds} around -2.9V", the output resistance is 26.7 MΩ, calculated by the following equation, "r_o=ΔV/ΔI". The output resistance is 22.3 MΩ in n-type mode (Supplementary Fig. 5c). When TG acting as PG, the output resistance values are 25.3 MΩ and 27.2 MΩ in p-type (Supplementary Fig. 5e) and n-type mode (Supplementary Fig. 5f), respectively. Besides, we calculated the intrinsic gain according the following equation, "A_v=g_m/g_{ds}"

[S19]. In this equation, $g_m=2I_d/(V_{gs}-V_{th})$, $g_{ds}=1/r_o$. When BG acting as PG, the intrinsic gain values are 85.71 and 53.70 in p-type (Supplementary Fig. 5b) and n-type mode (Supplementary Fig. 5c), respectively. When TG acting as PG, the intrinsic gain values are 68.06 and 27.74 in p-type (Supplementary Fig. 5e) and n-type mode (Supplementary Fig. 5f), respectively.

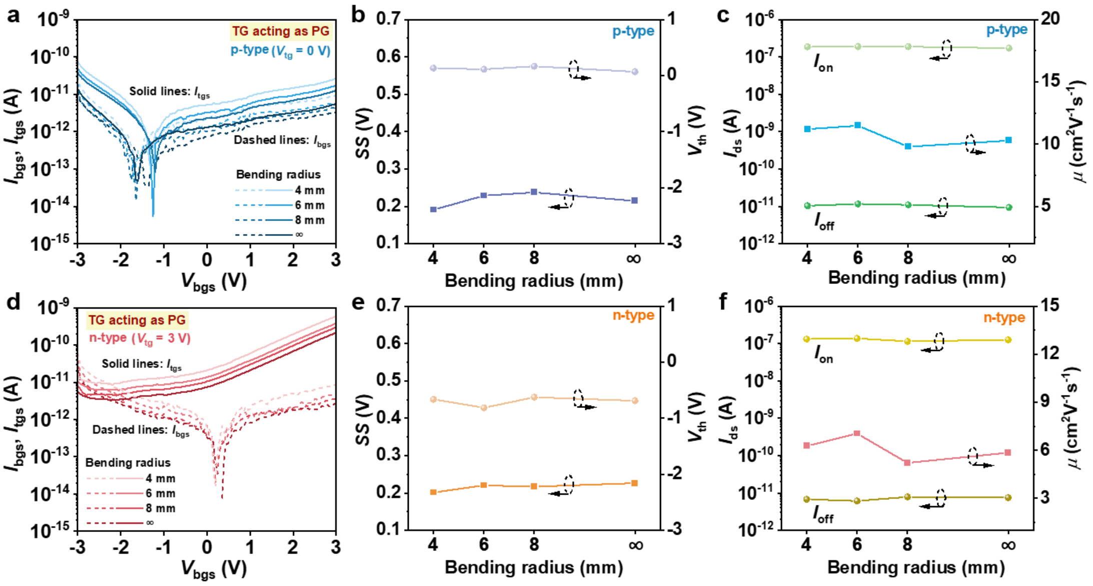

Supplementary Figure 6. Flexibility of IFMRT measured in situ when TG acting as PG. (a-c) Gate leakage current (a), V_{th} and SS (b), effective mobility, on-current and off-current (c) of the p-type IFMRT with the bending radius of 4 mm, 6 mm, 8 mm and ∞ (flat state). **(d-f)** Gate leakage current (d), V_{th} and SS (e), effective mobility, on-current and off-current (f) of the n-type IFMRT with the bending radius of 4 mm, 6 mm, 8 mm and ∞ (flat state).

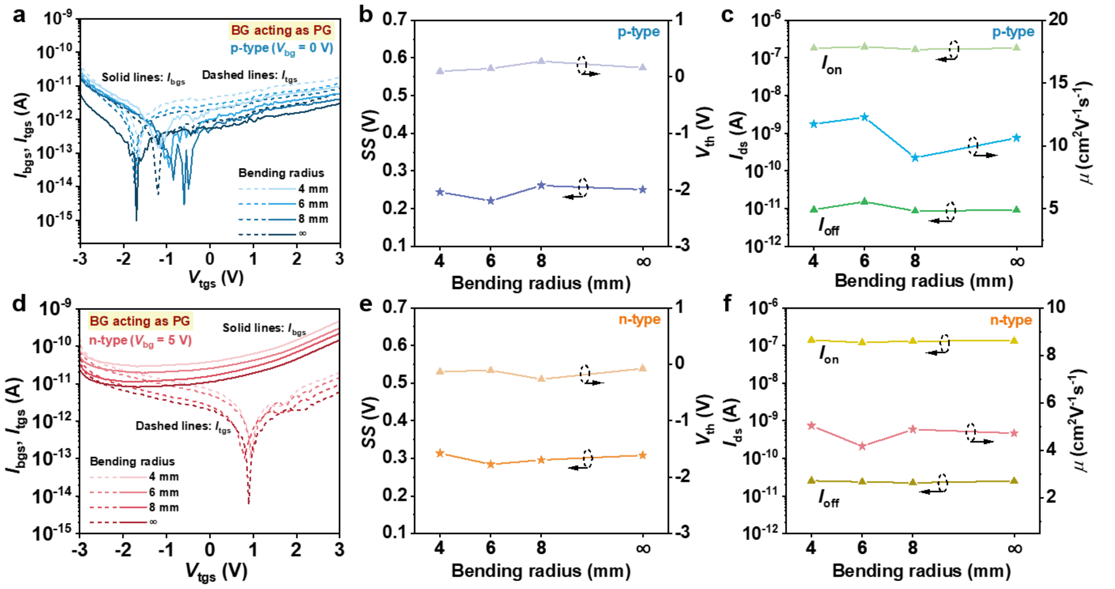

Supplementary Figure 7. Flexibility of IFMRT measured in situ when BG acting as PG. (a-c) Gate leakage current (a), V_{th} and SS (b), effective mobility, on-current and off-current (c) of the p-type IFMRT with the bending radius of 4 mm, 6 mm, 8 mm and ∞ (flat state). **(d-f)** Gate leakage current (d), V_{th} and SS (e), effective mobility, on-current and off-current (f) of the n-type IFMRT with the bending radius of 4 mm, 6 mm, 8 mm and ∞ (flat state).

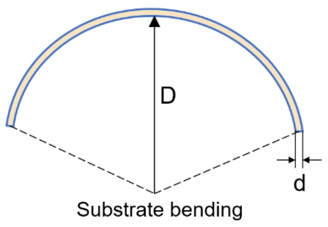

Supplementary Figure 8. Schematic diagram of the tensile strain calculation when bending the substrate.

Supplementary Note 6. The calculation of the tensile strain.

For thin flexible substrates under bending conditions, the tensile strain can be calculated by $d/2D$, where d is the substrate thickness and D is the bending radius, as illustrated in Supplementary Fig. 8. With our substrate thickness of 125 μm and bending radius of 4 mm, the calculated tensile strain is 1.6%.

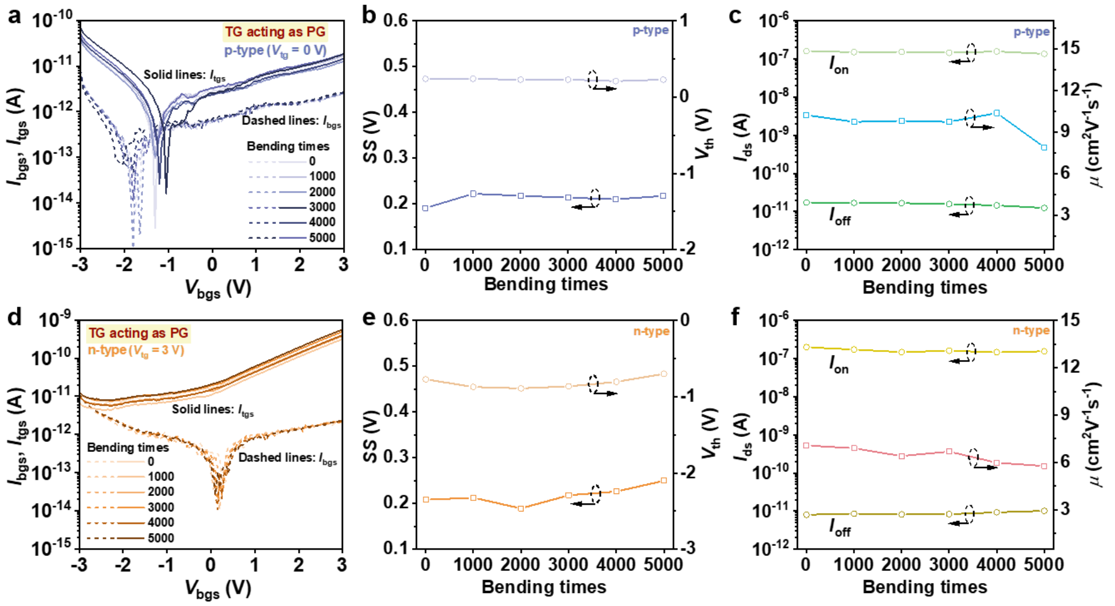

Supplementary Figure 9. Flexibility of IFMRT measured after different bending cycles when TG acting as PG. (a-c) Gate leakage current (a), V_{th} and SS (b), effective mobility, on-current and off-current (c) of the p-type IFMRT with the bending cycles from 0 to 5000. **(d-e)** Gate leakage current (d), V_{th} and SS (e), effective mobility, on-current and off-current (f) of the n-type IFMRT with the bending cycles from 0 to 5000.

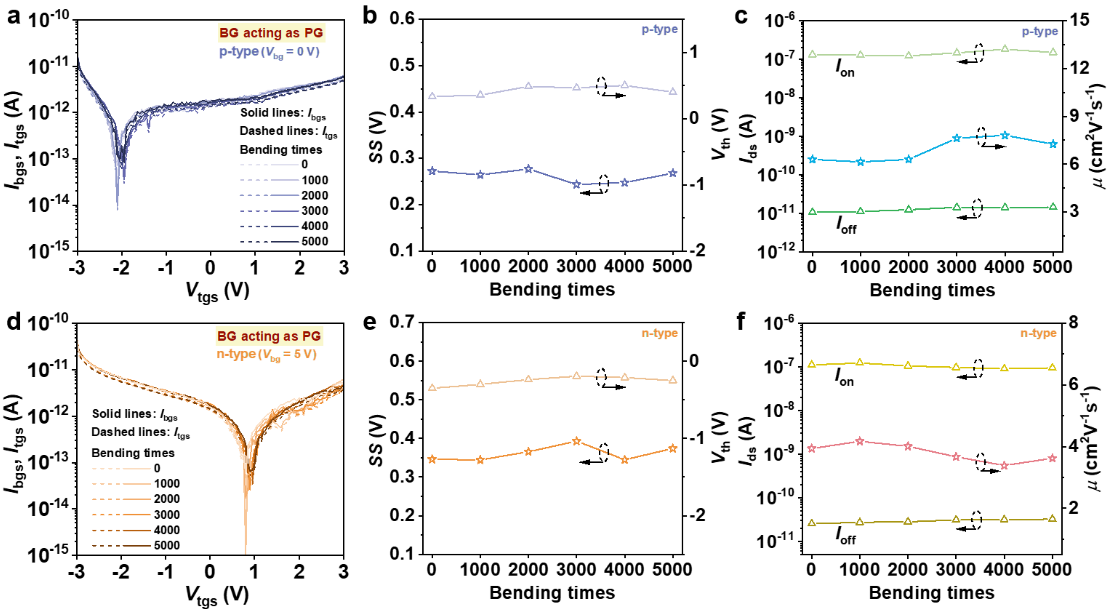

Supplementary Figure 10. Flexibility of IFMRT measured after different bending cycles when BG acting as PG. (a-c) Gate leakage current (a), V_{th} and *SS* (b), effective mobility, on-current and off-current (c) of the p-type IFMRT with the bending cycles from 0 to 5000. **(d-f)** Gate leakage current (d), V_{th} and *SS* (e), effective mobility, on-current and off-current (f) of the n-type IFMRT with the bending cycles from 0 to 5000.

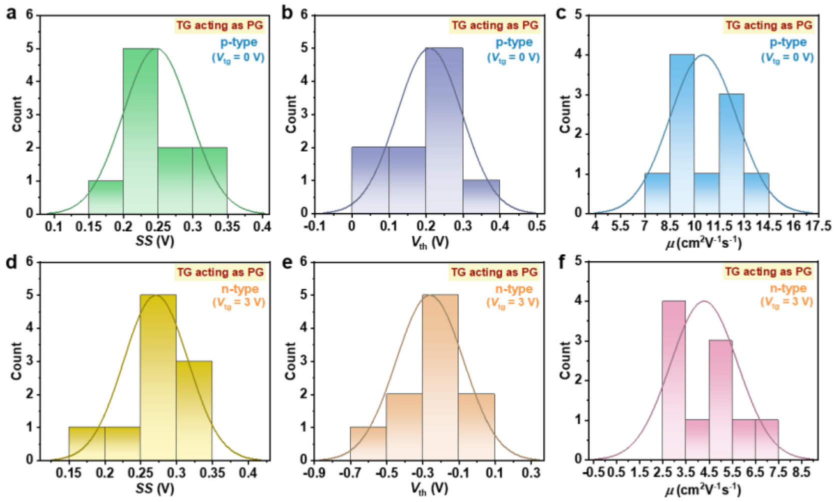

Supplementary Figure 11. Statistical data of IFMRTs when TG acting as PG. (a-c) The statistics of *SS* (a), V_{th} (b) and effective mobility (c) of p-type 10 IFMRTs. **(d-f)** The statistics of *SS* (d), V_{th} (e) and effective mobility (f) of n-type 10 IFMRTs.

Supplementary Note 7. The calculation of the effective mobility.

We calculate the saturation mobility according to the following formula:

$$\mu = \frac{2 I_{on} L}{C_{ox} W (V_{GS} - V_{th})^2} \quad (2)$$

C_{ox} is 125 nF·cm^{-2}, which is measured at a condition of "V_{cgs}=-3 V, V_{pgs}=0 V" and at a frequency of 335 Hz. V_{th} is extracted from the gate voltage value where the reverse-extrapolated line from the maximum transconductance point intersects the voltage axis.

Supplementary Note 8. The potential solution for the asymmetry of effective mobility.

The effective mobility asymmetry observed in Supplementary Fig. 11 can be attributed to the different Schottky barrier heights for electrons and holes at the metal-semiconductor contact. In this work, the slight p-type doping of S-CNTs results in a higher Schottky barrier height for electrons than for holes, which leads to a lower effective electron mobility. To achieve superior flexibility, we fabricated the electrodes with M-CNTs in this work. We are currently researching strategies to adjust the Fermi level of electrodes to achieve more balanced Schottky barrier heights for electrons and holes.

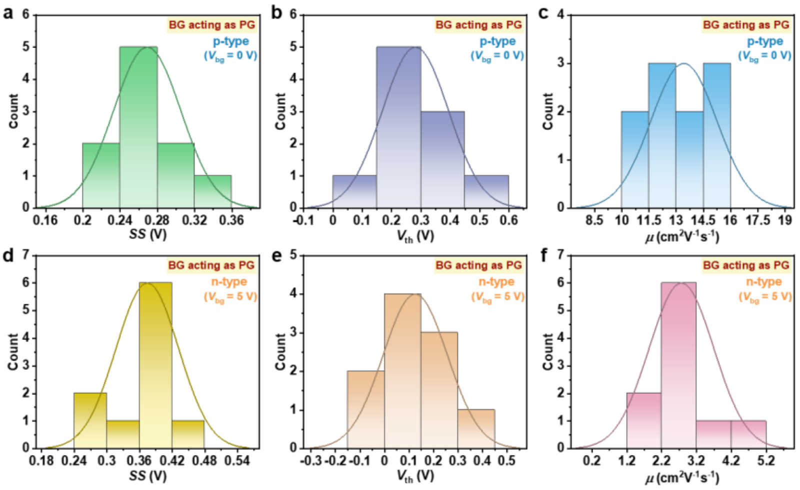

Supplementary Figure 12. Statistical data of IFMRTs when BG acting as PG. (a-c) The statistics of *SS* (a), V_{th} (b) and effective mobility (c) of p-type 10 IFMRTs. **(d-f)** The statistics of *SS* (d), V_{th} (e) and effective mobility (f) of n-type 10 IFMRTs.

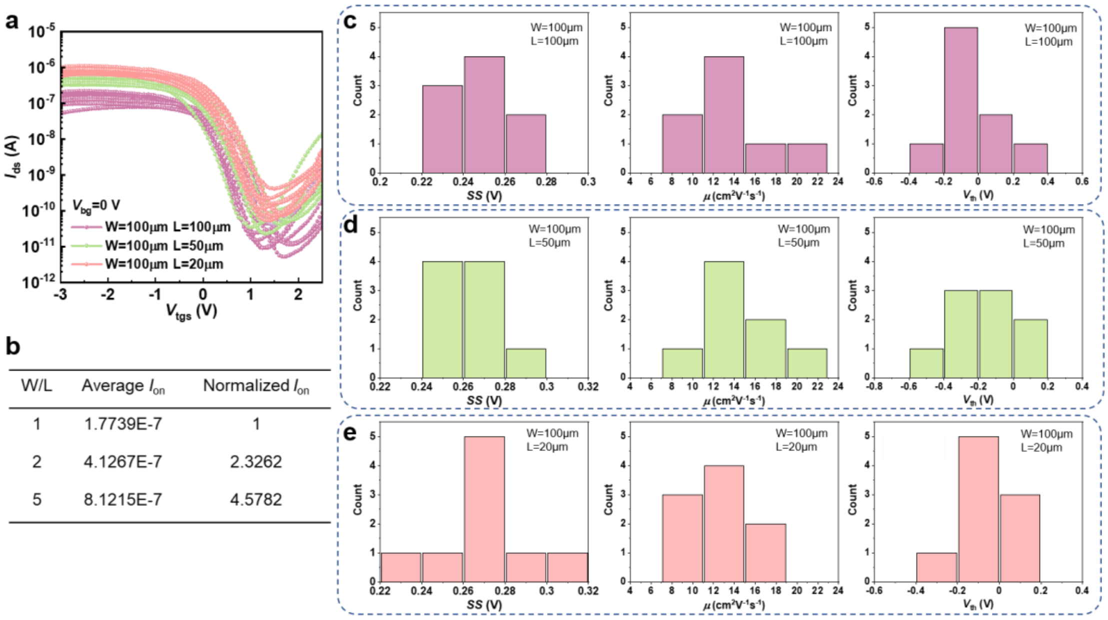

Supplementary Figure 13. Statistical data of p-type IFMRTs with three different channel sizes when BG acting as PG. (a) Transfer characteristics of p-type IFMRTs with three different channel sizes. (V_{ds}=−0.1 V) **(b)** Average I_{on} and normalized I_{on} of p-type IFMRTs with three different channel sizes. **(c-e)** The statistics of *SS*, effective mobility(μ) and V_{th} for p-type IFMRTs with W/L=1(c), W/L=2(d) and W/L=5(e).

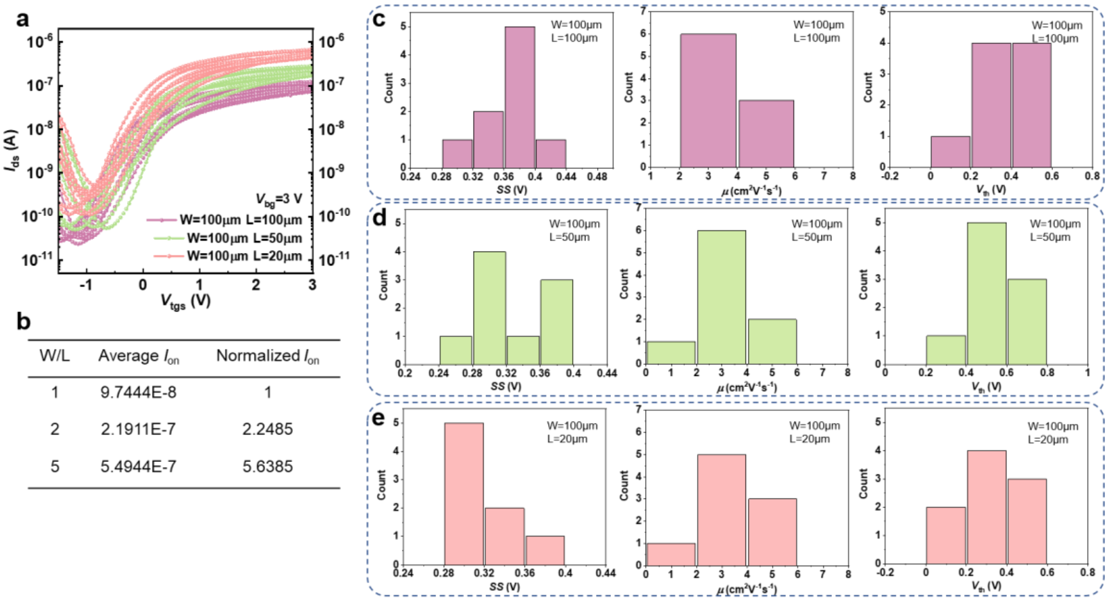

W/L	Average I_{on}	Normalized I_{on}
1	9.7444E-8	1
2	2.1911E-7	2.2485
5	5.4944E-7	5.6385

Supplementary Figure 14. Statistical data of n-type IFMRTs with three different channel sizes when BG acting as PG. (a) Transfer characteristics of n-type IFMRTs with three different channel sizes. (V_{ds}=−0.1 V) **(b)** Average I_{on} and normalized I_{on} of n-type IFMRTs with three different channel sizes. (**c-e**) The statistics of *SS*, effective mobility(μ) and V_{th} for n-type IFMRTs with W/L=1(c), W/L=2(d) and W/L=5(e).

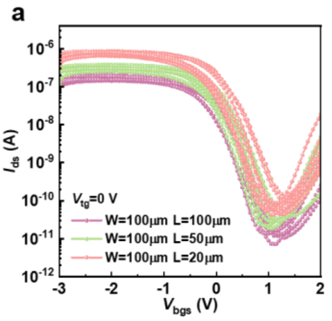

b

W/L	Average I_{on}	Normalized I_{on}
1	1.6970E-7	1
2	3.4093E-7	2.0089
5	7.8024E-7	4.5976

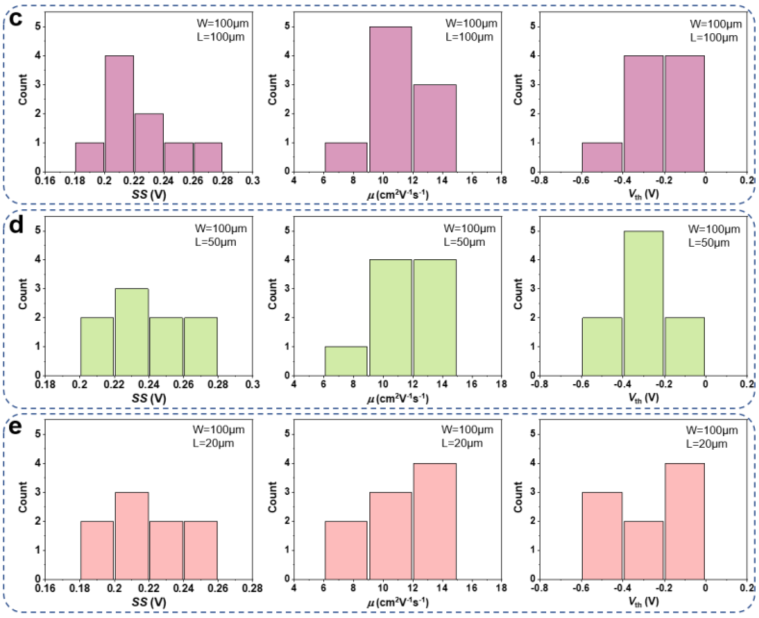

Supplementary Figure 15. Statistical data of p-type IFMRTs with three different channel sizes when TG acting as PG. (a) Transfer characteristics of p-type IFMRTs with three different channel sizes. (V_{ds}=−0.1 V) **(b)** Average I_{on} and normalized I_{on} of p-type IFMRTs with three different channel sizes. (**c-e**) The statistics of *SS*, effective mobility(μ) and V_{th} for p-type IFMRTs with W/L=1(c), W/L=2(d) and W/L=5(e).

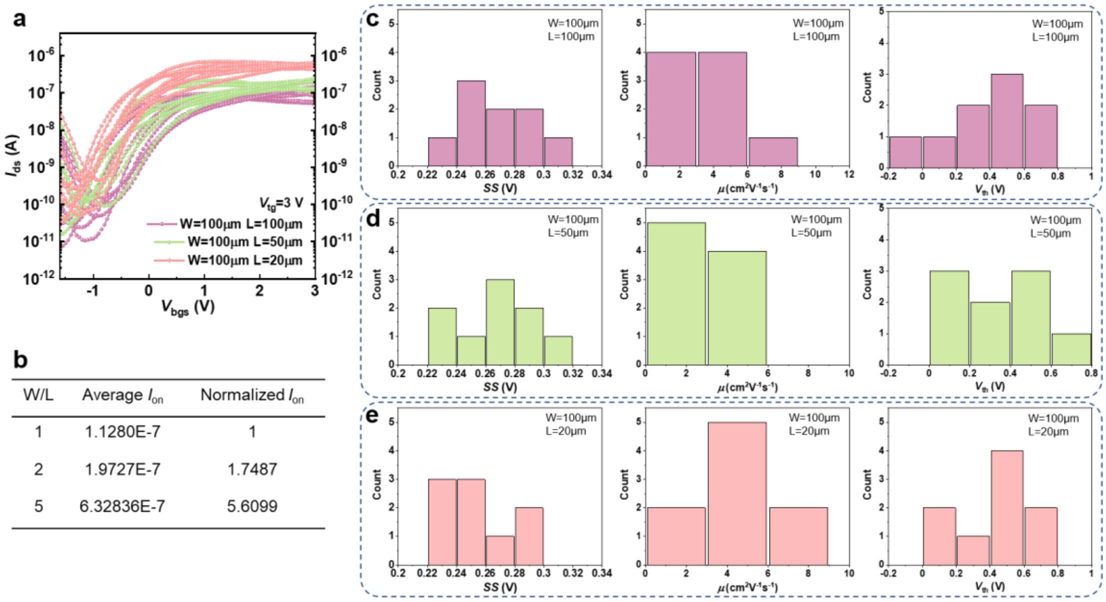

W/L	Average I_{on}	Normalized I_{on}
1	1.1280E-7	1
2	1.9727E-7	1.7487
5	6.32836E-7	5.6099

Supplementary Figure 16. Statistical data of n-type IFMRTs with three different channel sizes when TG acting as PG. (a) Transfer characteristics of n-type IFMRTs with three different channel sizes. (V_{ds}=−0.1 V) **(b)** Average I_{on} and normalized I_{on} of n-type IFMRTs with three different channel sizes. **(c-e)** The statistics of *SS*, effective mobility(μ) and V_{th} for n-type IFMRTs with W/L=1(c), W/L=2(d) and W/L=5(e).

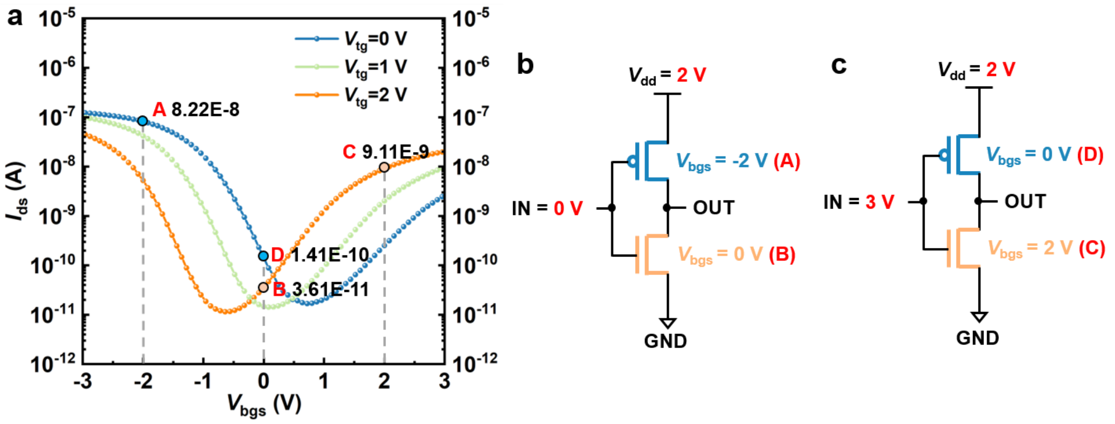

Supplementary Figure 17. Transistor operating point analysis in reconfigurable inverters when "Key=1". (a) The transfer characteristics of an IFMRT with reduced S-CNT under different PG voltages, marked with the corresponding operating points. (V_{ds}=−0.1 V, W=50 μm, L=50 μm) **(b-c)** Schematic diagram of an inverter when the input is 0 V (b) and 3 V (c).

Supplementary Note 9. The solution for short circuit conduction.

Adjusting the amount of S-CNTs in the channel can shift the whole curve left and decrease the current at "V_{bgs} = 0 V". Supplementary Fig. 17a shows the transfer characteristics of an IFMRT with reduced S-CNT under different PG voltages. Points A-D are consistent with the annotations in Supplementary Fig. 17b-c. For the initial p-type, when V_{tg} is 0 V (the blue curve), the current at "V_{bgs} = 0 V" is decreased to 1.41×10^{-10} A (point D). 2 V is enough for V_{tg} to modulate IFMRT to a proper n-type transistor (the orange curve), and the current at "V_{bgs} = 0 V" is 3.61×10^{-11} A (point B). The on-current of p-type and n-type IFMRTs (point A and C) are 8.22×10^{-8} A and 9.11×10^{-9} A. If constructing circuits based on this IFMRT, the short circuit conduction can be effectively mitigated, and the Key voltage (V_{dd} voltage) can be lowered to 2 V, as the IFMRT can be modulated into a proper n-type transistor without requiring high applied voltages. The fabrication process in a lab has limitations that introduce some device-to-device variations. For logic circuit applications, the amount of S-CNTs and current require precise control. We consider this a solvable challenge through engineering solutions.

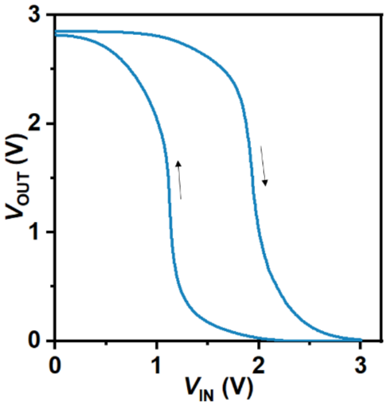

Supplementary Figure 18. The inverter exhibits a clockwise hysteresis behavior.

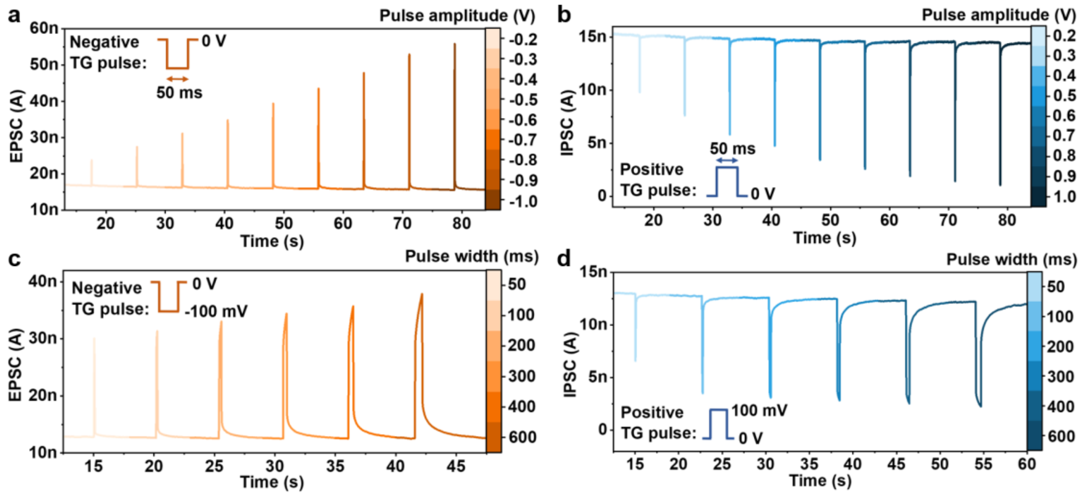

Supplementary Figure 19. Spike-voltage-dependent plasticity (SVDP) and the spike-duration-dependent plasticity (SDDP) of the artificial homo-synaptic device based on the IFMRT. (a-b) The excitatory postsynaptic current (EPSC) (a) and inhibitory postsynaptic current (IPSC) (b) triggered by presynaptic pulses with the identical width and different amplitudes. (V_{ds}=−0.1 V, W=50 μm, L=50 μm) **(c-d)** The EPSC (c) and IPSC (d) triggered by presynaptic pulses with the identical amplitude and different widths. (V_{ds}=−0.1 V, W=50 μm, L=50 μm)

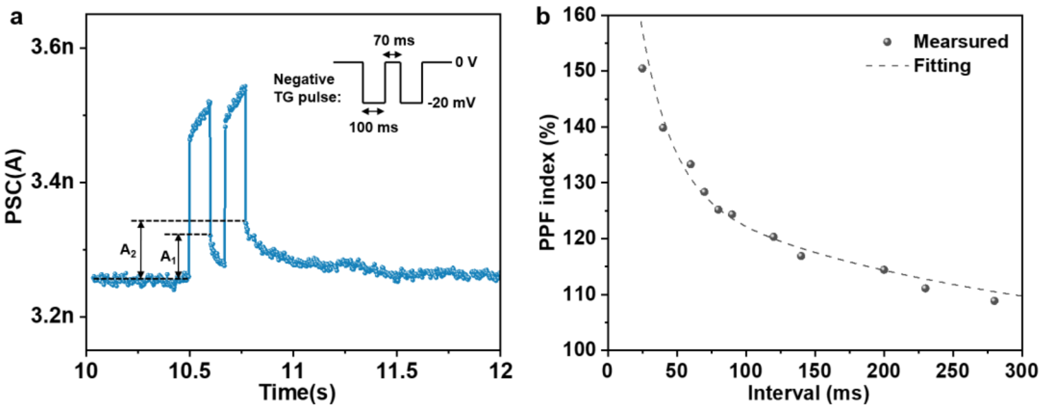

Supplementary Figure 20. PPF characteristics of the IFMRT. (a) PSC triggered by negative paired pulses separated by 70 ms (each pulse: -20 mV, 100 ms). (V_{ds}=−0.1 V, W=50 µm, L=50 µm) Negative paired pulses are applied to TG, and BG is fixed at 0 V. A_1 and A_2 are the current differences between the initial state and the pulse withdrawal. **(b)** PPF index ($A_2/A_1\times100\%$) varies with pulse interval.

Supplementary Note 10. Detailed descriptions of PPF.

The relationship between the pulse interval time and the PPF index in artificial synapses follows a double-exponential function:

$$PPF=C_1*\exp(-t/\tau_1)+C_2*\exp(-t/\tau_2)+C_0 \tag{3}$$

where C_0, C_1, C_2 are constants, t is the pulse interval time, and τ_1 and τ_2 are the relaxation time of short-term memory and long-term memory, respectively. According to the fitting results, τ 1 and τ2 are 22.4 ms and 253.5 ms respectively. τ_2 is about an order of magnitude greater than τ_1, which is consistent with the situation in biological synapses [S20].

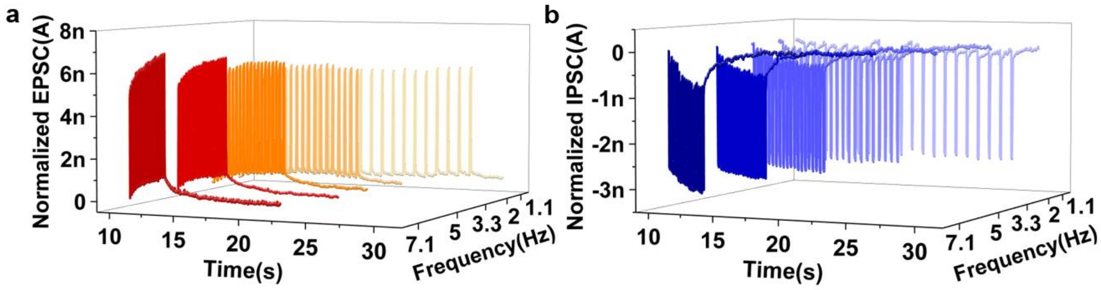

Supplementary Figure 21. Spike-frequency-dependent plasticity (SFDP) of the artificial homo-synaptic device based on the IFMRT. The normalized (a) EPSC and (b) IPSC triggered by 20 successive presynaptic pulses at different frequency with the pulse width of 100 ms and the pulse amplitude of -500 mV and 500 mV, respectively. (V_{ds}=−0.1 V, W=50 μm, L=50 μm)

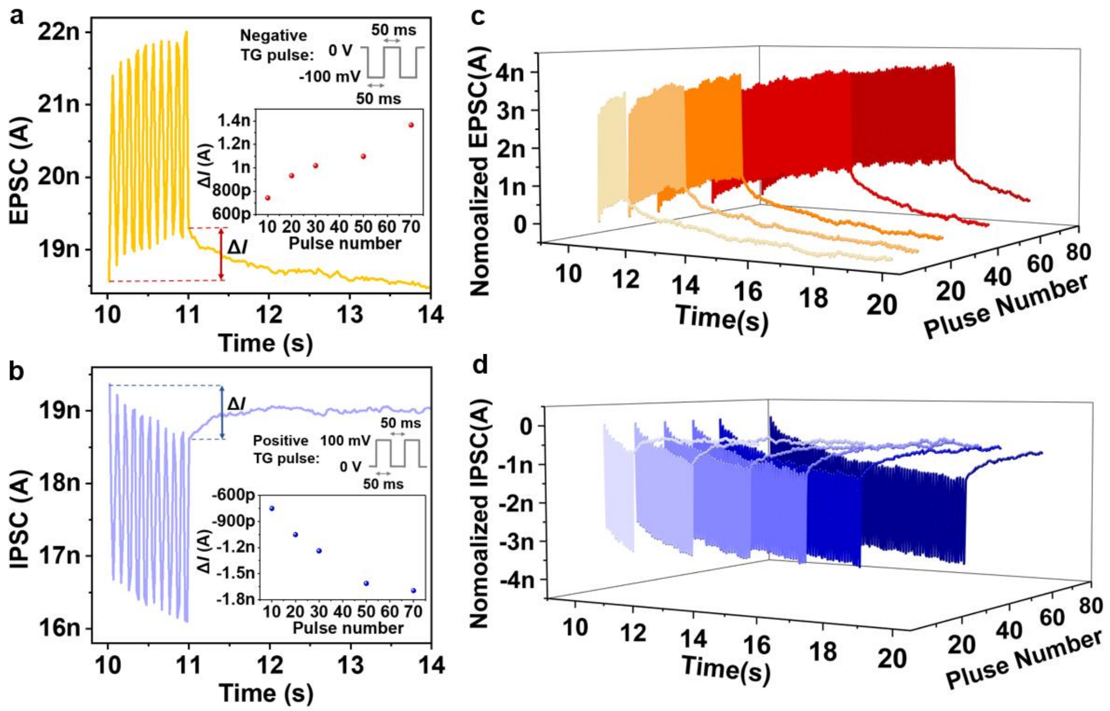

Supplementary Figure 22. Spike-number-dependent plasticity (SNDP) of the artificial homo-synaptic device based on the IFMRT. (a-b) EPSC (a) and IPSC (b) triggered by 10 negative and positive presynaptic spikes, respectively. (V_{ds}=−0.1 V, W=50 μm, L=50 μm) Inset: the relationship of ΔI and pulse number. **(c-d)** The normalized EPSC (c) and IPSC (d) triggered by negative and positive presynaptic spikes, respectively, with different pulse numbers from 10 to 70. (V_{ds}=−0.1 V, W=50 μm, L=50 μm)

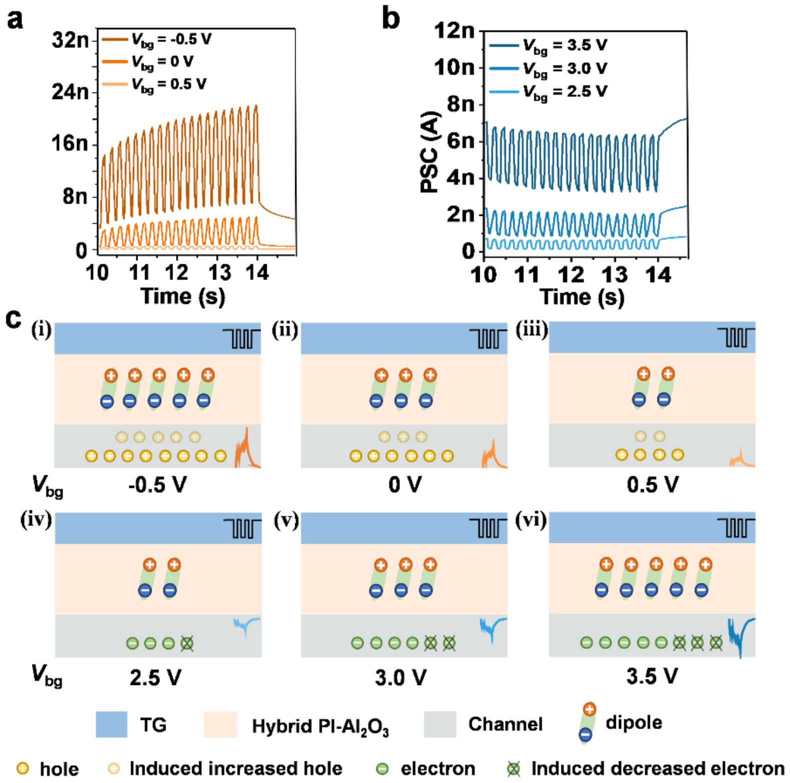

Supplementary Figure 23. The PSC and the schematic diagrams under different V_{bg} with negative pulses applied on TG. (a-b) PSCs in inhibitory mode (a) and excitatory mode (b) at different V_{bg}. (V_{ds}=−0.1 V, W=50 μm, L=50 μm) (20 negative presynaptic pulses with the pulse width of 100 ms and the pulse amplitude of -500 mV). **(c)** The schematic diagrams of the internal charges at different V_{bg}.

Supplementary References

S1 Peng, R. et al. Programmable graded doping for reconfigurable molybdenum ditelluride devices. *Nat. Electron.* **6**, 852-861 (2023).

S2 Zhou, Y. et al. Contact-engineered reconfigurable two-dimensional Schottky junction field-effect transistor with low leakage currents. *Nat. Commun.* **14**, 4270 (2023).

S3 Tsai, M. et al. A reconfigurable transistor and memory based on a two-dimensional heterostructure and photoinduced trapping. *Nat. Electron.* **6**, 755-764 (2023).

S4 Pan, C. et al. Reconfigurable logic and neuromorphic circuits based on electrically tunable two-dimensional homojunctions. *Nat. Electron.* **3**, 383-290 (2020).

S5 Sun, X. et al. Reconfigurable logic-in-memory architectures based on a two-dimensional van der Waals heterostructure device. *Nat. Electron.* **5**, 752–760 (2022).

S6 Ram, A. et al. Reconfigurable Multifunctional van der Waals Ferroelectric Devices and Logic Circuits. *ACS nano* **17**, 21865-21877 (2023).

S7 Li, X. et al. Cascaded Logic Gates Based on High-Performance Ambipolar Dual-Gate WSe2 Thin Film Transistors. *ACS nano* **17**, 12798-12808 (2023).

S8 Wu, P., Reis, D., Hu, X. S. & Appenzeller, J. Two-dimensional transistors with reconfigurable polarities for secure circuits. *Nat. Electron.* **4**, 45-53 (2021).

S9 Kang, J. et al. Non-Volatile Reconfigurable Four-Mode van der Waals Transistors and Transformable Logic Circuits. *ACS nano* **19**, 12948-12959 (2025).

S10 Deng, Q. et al. Reconfigurable Vertical Phototransistor with $MoTe_2$ Homojunction for High-Speed Rectifier and Multivalued Logical Circuits. *ACS nano* **18**, 23702-23710 (2024).

S11 Shim, H. et al. An elastic and reconfigurable synaptic transistor based on a stretchable bilayer semiconductor. *Nat. Electron.* **5**, 660-671 (2022).

S12 Huang, C., Zhang, Y. & Nomura, K. Reconfigurable Artificial Synapses with Excitatory and Inhibitory Response Enabled by an Ambipolar Oxide Thin-Film Transistor. *ACS Appl. Mater. Interfaces* **14**, 22252-22262 (2022).

S13 Yao, Y. et al. Reconfigurable Artificial Synapses between Excitatory and Inhibitory Modes Based on Single-Gate Graphene Transistors. *Adv. Electron. Mater.* **5**, 1800887 (2019).

S14 Duan, N. et al. Gate modulation of excitatory and inhibitory synaptic plasticity in a low-temperature polysilicon thin film synaptic transistor. *ACS Appl. Electron. Mater.* **1**, 132-140 (2018).

S15 Tian, H. et al. Graphene dynamic synapse with modulatable plasticity. *Nano lett.* **15**, 8013-8019 (2015).

S16 Kong, J. et al. Nanotube molecular wires as chemical sensors. *Science*, **287**, 622-625 (2000).

S17 Aguirre, C. M. et al. The Role of the Oxygen/Water Redox Couple in Suppressing Electron Conduction in Field-Effect Transistors. *Adv. Mater.* **21**, 3087-3091 (2009).

S18 Zhang, J., Wang, C., Fu, Y., He, Y. & Zhou, C. Air-Stable Conversion of Separated Carbon Nanotube Thin-Film Transistors from p-Type to n-Type Using Atomic Layer Deposition of High-kappa Oxide and Its Application in CMOS Logic Circuits. *ACS Nano*, **5**, 3284-3292 (2011).

S19 Long, G. et al. Super-saturated complementary carbon nanotube transistors with intrinsic gain singularities. *Nat. Commun.* **16**, 3390 (2025).

S20 Zucker, R. & Regehr, W. Short-term synaptic plasticity. *Annu. Rev. Physiol.* **64**, 355 (2002).
